# *miR-373* Suppresses Cell Proliferation and Apoptosis via Regulation of SIRT1/PGC-1α/NRF2 Axis in Pancreatic Cancer

**DOI:** 10.22074/cellj.2021.7038

**Published:** 2021-05-26

**Authors:** Qing-Hua Yin, Yuan Zhou, Zhi-Yuan Li

**Affiliations:** 1 Department of Hepatobiliary Surgery, The First Hospital of Changsha, Changsha 410000, P.R.China; 2 Department of Gastrointestinal Surgery, The Central Hospital of Hengyang City, Hengyang 421001, P.R.China

**Keywords:** *miR-373*, Oxidative Stress, Pancreatic Cancer, PGC-1α/NRF2 Pathway, SIRT1

## Abstract

**Objective:**

Our study aimed to investigate function and mechanism of *miR-373* in proliferation and apoptosis of pancreatic cancer (PC) cells by regulating NAD+-dependent histone deacetylase sirtulin 1 (SIRT1).

**Materials and Methods:**

This experimental study included two PC cell lines AsPC-1 and PANC-1 in which expression levels of *miR-373* and SIRT1 were manipulated. The level of *miR-373* was detected by reverse transcription quantitative polymerase chain reaction (RT-qPCR) method. Expression levels of SIRT1, BCL-2, BAX, cleaved CASPASE-8/9/3, PARP, PGC-1α, NRF2, eNOS and iNOS were examined via RT-qPCR and western blotting, respectively. The binding sites of *miR-373* on the SIRT1 were examined via dual-luciferase assay. Cell proliferation and apoptosis were examined by MTT assay, colony formation assay, Annexin-V/PI staining and TUNEL assay. The oxidative metabolic changes were monitored by reactive oxygen species (ROS), malondialdehyde (MDA) and superoxide dismutase (SOD) detection.

**Results:**

*miR-373* could specifically target the 3’-UTR of SIRT1 and reduce its expression in PC cells. Either elevated expression of *miR-373* or partial loss of SIRT1 inhibited cell proliferation and induced cell apoptosis. Accumulation of BAX and cleaved CASPASE-8/9/3, inhibition of PGC-1α/NRF2 pathway, increase oxidative stress and reduction of BCL-2 as well as uncleaved PARP were found in the presence of *miR-373* or the absence of SIRT1. Overexpression of SIRT1 could reduce anti-proliferative and pro-apoptotic effects of *miR-373*.

**Conclusion:**

Overall, this study concluded that *miR-373*-dependent SIRT1 inhibition displays anti-proliferative and pro- apoptotic roles in PC cells via PGC-1α/NRF2 pathway, which highlights *miR-373* as a potential target for PC treatment.

## Introduction

Pancreatic cancer (PC) is a notoriously fatal malignant tumor, characterized by a highly aggressive potential of invasion and metastasis (1, 2). At present, PC ranks the 4^th^ cause of cancer related death, with statistics suggesting that it will eventually become the 2^nd^ lethal cancer within the next decade (3). Owing to the lack of incipient symptom, PC patients are often diagnosed with a high-grade stage (4). Neo-adjuvant treatment in combination with radiotherapy/chemotherapy at present represents the best chance of increasing overall survival of patients (5). However, local recurrence and cancer metastasis to other organs suggest frequent issues faced during the treatment of PC (6). Therefore, it is urgent to explore new therapeutic strategies for PC treatment.

microRNAs (miRNAs), as a group of small non-coding RNAs, can bind to target genes and suppress their expressions (7, 8). Over the last decade, miRNAs have been highlighted due to their crucial roles in the molecular processes involved in the initiation and progression of various tumor types (9-12). For instance, *miR-373* was shown to be dramatically down-regulated in PC, and this decline of **miR-373** expression facilitated invasion of cancer cells by enhancing epithelial-mesenchymal transition (13). In line with this work, down-regulation of *miR-373* was also proved in the serum of PC patients, suggesting *miR-373* might serve as an independent predictor for early detection and prognosis of PC (14). It was reported that *miR-373* targeted Cycin D2 (CCND2) to promote the chemosensitivity of gemcitabine via cell cycle pathway in pancreatic carcinoma cells (15). *miR-373* could mediate qingyihuaji formula (QYHJ) effect on reversing gemcitabine-triggered resistant of human PC (16). However, the downstream regulatory mechanism of *miR-373*-mediated PC progression is still rarely reported. 

Silent mating-type information regulation 2 homolog (SIRT1) is an NAD^+^ -dependent class III histone deacetylase, which has key roles in metabolic control (17). Increasing studies showed that SIRT1 was induced in PC and involved in the regulating proliferation and viability of PC cells. Recently, *miR-373* was proved as a potential therapeutic strategy for renal fibrosis by playing regulatory role in modulating SIRT1-mediated NF-κB/ MMP-9 signaling (18). However, study of the relationship between *SIRT1* and *miR-373* in PC progression is yet unclear. SIRT1 participates in mitochondrial biogenesis to maintain cellular redox homeostasis by deacetylation of PGC-1α, which is a peroxisome proliferator-activated metabolic regulator (19). The shear stress-induced SIRT1 could initiate PGC-1α production and activation, and thus it could enhance mitochondrial biogenesis (20). Nuclear factor E2-related factor 2 (NRF2), as an important cellular oxidative stress regulatory transcript factor, is an effective drug target for antioxidant therapy (21). NRF2 is transported to nucleus and induces production of downstream detoxification as well as antioxidant enzymes to produce an antioxidant effect in response to oxidative stresses (22, 23). SIRT1 was revealed to promote activity of NRF2 and increase expression levels of the NRF2 downstream genes (24). Hitherto, the role of *miR-373* on proliferation and apoptosis of PC cells via modulation of PGC-1α/NRF2 pathway is a mystery.

Presently, the study of *miR-373* regulated SIRT1 in PC is still lacking and the roles of this regulatory axis in the PC progression are unclear. Therefore, based the previous studies, the crucial objective of this study is to investigate *miR-373*-mediated SIRT1 regulation and its roles in the growth and progression of PC. Hopefully, this work can shed some light on finding alternative therapy method for PC and enrich the theoretical bases of PC treatment. 

## Materials and Methods

### Cell culture

In this experimental study, PC cell lines AsPC-1 and PANC-1 were obtained from American Type Culture Collection (ATCC, USA). PC cells were cultured in Dulbecco’s Modified Eagle’s Medium (DMEM, Sigma-Aldrich, USA) supplemented with 10% fetal bovine serum and 100 U/mL penicillin/streptomycin (Thermo Fisher Scientific, USA) in a humidified 37˚C incubator with 5% CO_2_ . Further experiments were performed once cell confluence reached 70-80%. 

### Cell grouping and transfection

PC cells were trypsinized and inoculated into 24-well plates. After removing culture medium, the cells were classified into the following groups: i. *miR-373* negative control (NC) group (cells were treated with NC for *miR-373* mimics), ii. *miR-373* mimics group (cells were treated with *miR-373* mimics to elevate expression of *miR-373*), iii. shNC group (cells were treated with NC for shRNA as negative control), iv. sh*SIRT1* group (cells were treated with shRNA-*SIRT1* to knockdown *SIRT1*), v. *miR-373* mimics+pcDNA3.1-NC (cells were treated with *miR-373* mimics followed by the pcDNA3.1 empty vector), and vi. *miR-373* mimics+pcDNA3.1-*SIRT1* (cells were treated with *miR-373* mimics followed by pcDNA3.1- *SIRT1* vector). The cells were transfected for 48 hours as above indicated, according to the guidelines for the Lipofectamine™ 2000 (Invitrogen, USA). *miR-373* NC, *miR-373* mimics, shNC, sh*SIRT1*, pcDNA3.1-NC and pcDNA3.1-*SIRT1* were all purchased from GenePharma (Shanghai, China).

### Dual-luciferase reporter gene assay

Binding sites between *miR-373* and *SIRT1* were predicted based on a bioinformatics prediction website (http://mirtarbase.mbc.nctu.edu.tw/php/index.php). The fragment sequences involved at the site of action were obtained. Dual-luciferase reporter gene assay was adopted to detect the relationship between *miR-373* and *SIRT1* and to identify whether *SIRT1* was indeed a direct target gene of *miR-373*. According to the binding sequences of 3´- UTR of *SIRT1*, both the wild type and mutation sequences were designed and synthesized accordingly from Sangon Biotech (Shanghai, China). *SIRT1 *3´-UTR was cloned into pGL4 luciferase reporter plasmid (Promega, USA). Cells were co-transfected with pGL4-*SIRT1* or control pGL4 reporter plasmid and *miR-373* mimics for 48 hours by Lipofectamine™ 2000 (Invitrogen, USA). Changes in the luciferase activity among the groups were detected using a dual-luciferase detection kit (D0010; Beijing Solarbio Science & Technology Co. Ltd., China). The fluorescence intensities were observed by GLomax20/20 Luminometer (E5311; Shaanxi Zhongmei Biotechnology Co., China).

### MTT assay

MTT assay was performed to test cell proliferation. Transfected PC cells were seeded into 96-well plates and then incubated for 12 hours. 20 μl of MTT (Sigma-Aldrich, USA) was added into 96-well plates and incubated for 4 hours in a humidified 37˚C incubator with 5% CO_2_ . Then, medium was removed and 150 μl dimethyl sulfoxide (DMSO, Sigma-Aldrich, USA) was added into 96-well plates. Finally, following shaking at 25˚C for 15 minutes, the optical density was measured at 490 nm by microplate reader (Bio-Rad Laboratories Inc., USA).

### Colony formation assay

PC cells were seeded in 6-well plates with 500 cells/ well and cultured in a humidified 37˚C incubator with 5% CO_2_ for 2 weeks. The cells were washed with phosphate buffer saline (PBS, Sigma-Aldrich, USA) and then fixed by 70% ethanol for 3 minutes after formation of clear colonies. The cells were then stained for 30 minutes with 1% crystal violet (Beyotime, China). The colony number was counted under the microscope.

### Apoptosis assay

Apoptotic cells were observed via Annexin V-FITC Apoptosis Detection Kit (Sigma-Aldrich, USA) following the manufacturer’s directions. Cells were collected and then washed with pre-cold PBS for twice and resuspended in 1× binding buffer. The cells were labeled with 5 μl Annexin V-FITC for 15 minutes and then 5 μl of PI for 10 minutes at 25˚C in dark. The cells were checked through a FACSCanto II flow cytometer (BD Biosciences, Germany).

### TUNEL assay

Cleavage of genomic DNA during apoptosis was measured with In Situ Apoptosis Detection kit from KeyGen BioTech Ltd. (Jiangsu, China). The samples were rinsed for 3 times with PBS solution. Then, 100 μl Proteinase K solution (9:1 mixture of PBS and Proteinase K) was added at 37˚C for 30 minutes and washed with PBS for 3 times. In the following step, 50 μl TdT enzyme solution was added into the cell samples and incubated at 37˚C for 60 minutes in dark. After washing with PBS for 3 times, the samples were supplemented with 5 μl Streptavidin-Fluorescein solution and 45 μl Labeling Buffer, and incubated at 37˚C for 30 minutes in dark. After washing with PBS and staining with 4’,6-diamidino-2- phenylindole (DAPI, Sigma-Aldrich, USA) the cells were observed and photographed via fluorescence microscope.

### Analysis of oxidative stress indicators

Cells were lysed with Tris-HCl (Beijing Biotopped Science & Technology Co., China) and treated with extraction buffer (150 mM NaCl, 1 mM Na_2_ EDTA, 1 mM EGTA, 2.5 mM sodium pyrophosphate and 1% NP-40) on ice. The cells were then centrifuged at 12,000 rpm at 4˚C for 10 minutes. The cell lysates were used for further reactive oxygen species (ROS), malondialdehyde (MDA) and superoxide dismutase (SOD) measurement. ROS, MDA and SOD values of PC cells were detected using the kits produced by Nanjing Jiangcheng Bioengineering Institute (China), and measurement was conducted according to the protocols provided by the manufacturer.

### RNA extraction and reverse transcription quantitative polymerase chain reaction 

Total RNA was extracted from PC cells using Trizol Kit (ThermoFisher Scientific, USA) based on the manufacturer’s instruction, followed by measurement of RNA concentration. The primers were synthesized from TaKaRa Biotechnology Co. (China). Sequences of PCR primers were as follows:

*miR-373*-

F: 5ˊ-GTAGCAGGATGGCCCTAGAC-3ˊ

R: 5ˊ-CGCCCTCTGAACCTTCTCTT-3ˊ

*SIRT1-*

F: 5ˊ-TAGCCTTGTCAGATAAGGAAGGA-3ˊ 

R: 5ˊ-ACAGCTTCACAGTCAACTTTGT-3ˊ

*U6* snRNA-

F: 5ˊ-AAAGCAAATCATCGGACGACC-3ˊ

R: 5ˊ-GTACAACACATTGTTTCCTCGGA-3ˊ

*GAPDH*-

F: 5ˊ-GTCGGAGTCAACGGATTTGG-3ˊ

R: 5′-AAAAGCAGCCCTGGTGACC-3ˊ

Reverse transcription was conducted by the PrimeScript RT reagent Kit (TaKaRa Biotechnology Co.). The obtained cDNA was diluted to 50 ng/μl to perform reverse transcription quantitative polymerase chain reaction (RT-qPCR) with SYBR Premix EX TaqTM kit (TaKaRa Biotechnology Co.) in an ABI 7500HT real time PCR system (Applied Biosystems, USA). Glyceraldehyde phosphate dehydrogenase (*GAPDH*) and *U6* snRNA were used as internal controls for mRNA and miRNA, respectively. Relative expression levels were measured by the 2^-ΔΔCq^ method.

### Western blotting assay

The cells were harvested and lysed with RIPA buffer (Sigma-Aldrich, USA). Protein concentration of each sample was evaluated by bicinchoninic acid (BCA) protein assay kit (Yi Sheng Biotechnology Co., China). After separation by SDS-PAGE, the proteins were transferred onto polyvinylidenefluoride (PVDF) membranes, and sealed with 5% bovine serum albumin (BSA) at room temperature for 1 hour. The membranes were incubated with primary antibodies SIRT1, BAX, BCL-2, CASPASE-9, CASPASE-8, CASPASE-3, PARP, PGC-1α, NRF2, eNOS, iNOS and GAPDH for 12 hours at 4°C. Then, the membranes were washed three times with Tris-buffered saline containing 0.1% Tween 20 (TBST). Diluted horseradish peroxidase (HRP)-labeled goat anti-rabbit secondary antibody was added to the samples at 25˚C for 1 hour. The membranes were again washed three times with TBST, and the protein bands were visualized using Immobilon Western Chemiluminescent HRP substrate (Millipore, USA). Quantitative protein analysis was performed by ImageJ 1.48u software (National Institutes of Health, USA). GAPDH was served as the internal reference. The antibodies for western blotting were produced by Cell Signaling Technology (USA).

### Statistical analysis

All experiments were performed at least for three times in triplicate. Data were expressed as mean ± standard deviation (SD). Data were analyzed with Prism 6.0 (GraphPad Software, USA). Statistical evaluation was performed via Student’s t test between two groups and one-way analysis of variance (ANOVA) followed by Tukey’s post hoc test for multiple comparisons. Value of P<0.05 was statistically considered significant.

## Results

### *miR-373* negatively regulates SIRT1 expression level by binding to *SIRT1 3´*-UTR directly

**Fig.1:**
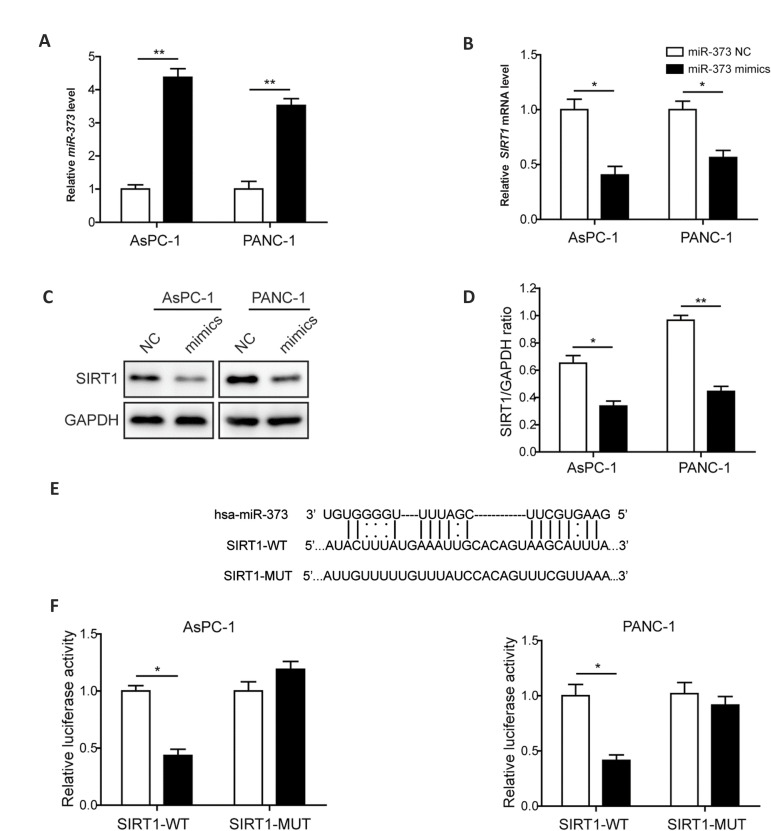
*miR-373* negatively regulates SIRT1 expression level by binding to *SIRT1* 3’-UTR directly. **A. **Relative expression level of *miR-373* in control and mimics-transfected PC cell lines. **B.** Expression of *SIRT1* evaluated by RT-qPCR under regulation of *miR-373*. **C. **Protein level of SIRT1 in *miR-373* mimics-transfected PC cell lines and control cells. **D.** Statistical analysis of the relative protein expression of SIRT1 in the presence of *miR-373* overexpression. **E.** The binding sequences between 3’-UTR of *SIRT1* and *miR-373*. **F. **Luciferase activity detection. PC; Pancreatic cancer, RT-PCR; Reverse transcriptionpolymerase chain reaction,*; P<0.05, and **; P<0.01.

The expression of *miR-373* was monitored by RT-qPCR and the results showed that in comparison with the *miR-373* NC treated group, level of the *miR-373* was boosted for about 3-4 times after *miR-373* mimics treatment in the both AsPC-1 and PANC-1 cells (Fig .1A). This result suggested successful introduction of *miR-373* mimics into the PC cells included in this study. To test whether *miR-373* can regulate *SIRT1* related to proliferation and apoptosis of PC cells, expression of SIRT1 was monitored upon the application of *miR-373* mimics. As indicated in Figure 1B, mRNA level of *SIRT1* was suppressed by overexpression of *miR-373* in the AsPC-1 and PANC-1 cells compared to *miR-373* NC treated group. Expression of SIRT1 was also monitored at protein level by western blotting. Result revealed that accumulation of SIRT1 protein in tested PC cell lines was decreased about 50% upon overexpression of *miR-373* (Fig .1C, D). These results suggested the *miR-373* suppressed expression of SIRT1 in PC cells.

As *miR-373* was able to down-regulate the expression of *SIRT1*, the potential target 3´-UTR sequence of *miR-373* in *SIRT1* was predicted (Fig .1E), and dualluciferase reporter assay was employed to identify the potential interaction. As shown in Figure 1F, the relative luciferase activity was declined significantly in AsPC-1 and PANC-1 cells co-transfected with *miR-373* mimics and constructs harboring wild-type of *SIRT1* 3´-UTR. However, luciferase activity retained similar to control in the cells transfected with *miR-373* mimics and constructs harboring *SIRT1* 3´-UTR with mutation at the predicted seed binding sites (Fig .1F). According to these results, we concluded that *miR-373* was able to suppress SIRT1 expression by direct targeting.

### Restoring *miR-373* or silencing *SIRT1* inhibits proliferation of pancreatic cancer cells

**Fig.2:**
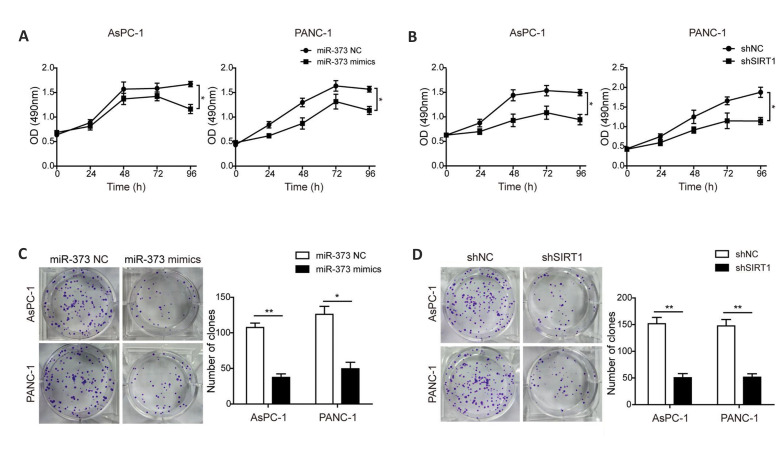
*miR-373* and *SIRT1* regulate proliferation of PC cells. Proliferation of the AsPC-1 and PANC-1 cells after transfection of **A.** *miR-373* mimics and **B.** sh*SIRT1* were examined by MTT assay. Colony formation of the AsPC-1 and PANC-1 cells after transfection of** C.** *miR-373* mimics and **D.** sh*SIRT1* were evaluated by colony formation analysis. PC; Pancreatic cancer, h; Hour, *; P<0.05, and **; P<0.01.

To study the regulatory functions of *miR-373* and *SIRT1* in proliferation of PC cells, MTT and colony formation assays were conducted. The results indicated that introduction of *miR-373* mimics and sh*SIRT1 *were both able to hinder proliferation of PC cells (Fig.2A, B). Additionally, as presented in Figure 2C and 2D, transfection with *miR-373* mimics and sh*SIRT1* both impaired colony formation ability of the AsPC-1 and PANC-1 cells versus control group. These results indicated that overexpression of *miR-373* or knockdown of *SIRT1* could dispute proliferation of PC cells. 

### Restoring *miR-373* or silencing *SIRT1* facilitates apoptosis of pancreatic cancer cells

**Fig.3:**
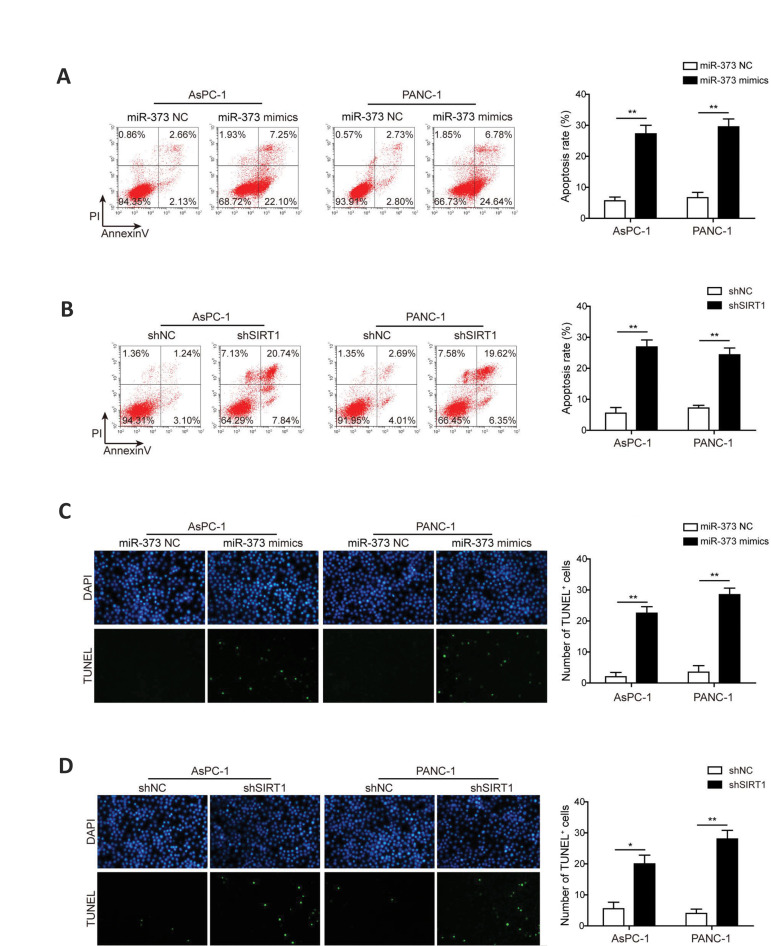
Effects *miR-373* and *SIRT1* on apoptosis of PC cells. **A.** Detection of cell apoptosis by flow cytometry in *miR-373* mimics-transfected AsPC-1 and PANC-1 cells. **B.** Detection of cell apoptosis by flow cytometry in AsPC-1 and PANC-1 cells after *SIRT1* knockdown. **C. **Representative pictures of apoptosis detection by TUNEL in *miR-373* mimics-transfected AsPC-1 and PANC-1 cells. **D. **Representative pictures of apoptosis detection by TUNEL assay in sh*SIRT1*- transfected AsPC-1 and PANC-1 cells. PC; Pancreatic cancer, *; P<0.05, and **; P<0.01.

To explore the effects of *miR-373* and *SIRT1* on cell apoptosis, the PC cells transfected with *miR-373* mimics or sh*SIRT1* were analyzed. The data collected using flow cytometry revealed that the number of apoptotic PC cells in the both *miR-373* mimics-transfected cells was increased significantly in comparison with the control groups (Fig .3A). Similarly, transfection of sh*SIRT1* increased apoptosis ratio of PC cells in the two cell lines included in this study (Fig .3B). Furthermore, TUNEL staining consistently showed that introduction of either *miR-373* mimics or sh*SIRT1* could significantly promote the number of TUNEL^+^ cells in the both AsPC-1 and PANC-1 cells (Fig .3C, D). All together, these findings indicated that overexpression of *miR-373* or silence of *SIRT1* enhanced apoptosis of PC cells.

### Restoring *miR-373* or silencing *SIRT1* regulates apoptosis-related proteins in pancreatic cancer cells

**Fig.4:**
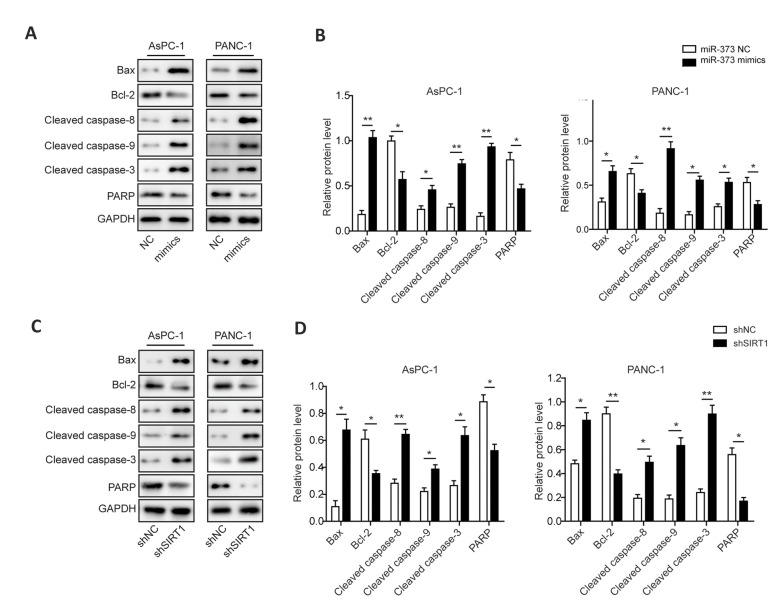
*miR-373* and *SIRT1* regulate apoptosis-associated proteins in PC cells. **A.** Protein expressions of BAX, BCL-2, cleaved CASPASE-8/9/3 and PARP in *miR-373* mimics-transfected PC cells. **B. **Statistical analysis of the relative protein expressions of BAX, BCL-2, cleaved CASPASE-8/9/3 and PARP in the presence of *miR-373* overexpression. **C. **Protein expressions of BAX, BCL-2, cleaved CASPASE-8/9/3 and PARP in sh*SIRT1*-transfected PC cells. **D.** Statistical analysis of the relative protein expressions of BAX, BCL-2, cleaved CASPASE-8/9/3 and PARP in sh*SIRT1*-transfected PC cells. PC; Pancreatic cancer, *; P<0.05, and **; P<0.01.

To further study the cellular mechanisms of proliferation and apoptosis alteration in *miR-373* mimics and sh*SIRT1*-transfected PC cells, western blot was performed to examine the expression levels of apoptosis-associated proteins. Introduction of *miR-373* mimics significantly increased accumulation of BAX and cleaved CASPASE-8/9/3 in the two PC cell lines included in this work, while the expression levels of BCL-2 and uncleaved PARP were suppressed (Fig .4A, B). Likewise, silence of *SIRT1* also promoted expression of BAX and activated CASPASE-8/9/3 in studied PC cells, whereas the accumulation of BCL-2 and uncleaved PARP were declined (Fig .4C, D). These results indicated that *miR-373* and *SIRT1*-mediated cell apoptosis was mediated by activating CASPASE-8/9/3 signaling pathways in PC cells.

### Restoring *miR-373* or silencing *SIRT1* inhibits PGC1α/Nrf2 signaling pathway and improves oxidative stress response in pancreatic cancer cells

**Fig.5:**
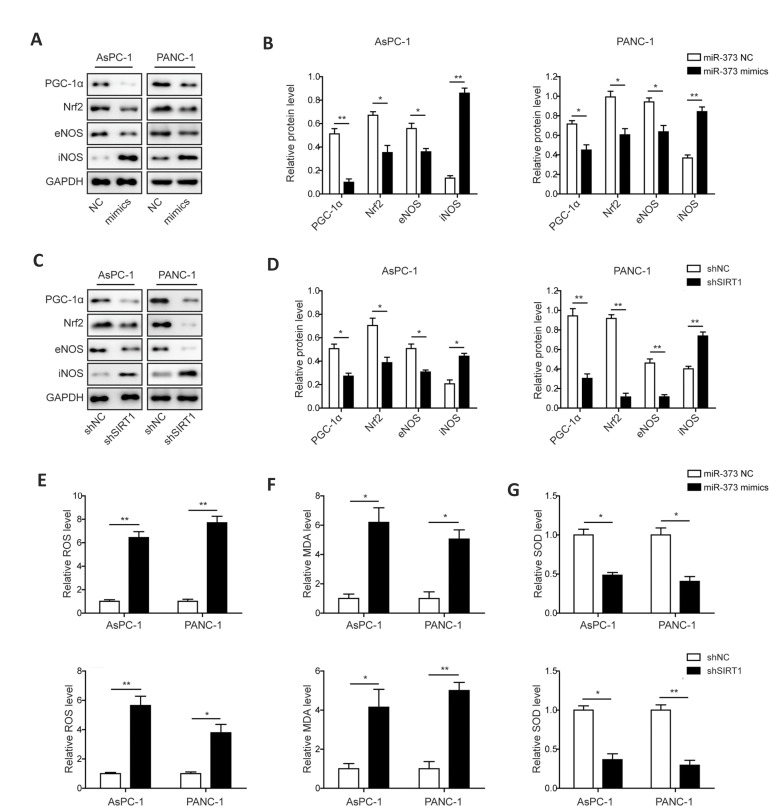
Effects of *miR-373* and *SIRT1* on PGC-1α/NRF2 pathway and oxidative stress response in PC cells. **A.** Levels of PGC-1α, NRF2, eNOS and iNOS in *miR-373* mimics-transfected PC cells. **B.** Statistical analysis of the relative protein levels of PGC-1α, NRF2, eNOS and iNOS in the presence of *miR-373* overexpression. **C.** Expression levels of PGC-1α, NRF2, eNOS and iNOS in shSIRT1-transfected PC cells. **D. **Statistical analysis of the relative protein expression of PGC-1α, NRF2, eNOS and iNOS in the presence of sh*SIRT1*. **E.** Relative ROS level in *miR-373* mimics or sh*SIRT1*-transfected PC cells. **F. **Relative MDA level in *miR-373* mimics or sh*SIRT1*-transfected PC cells. **G.** Relative SOD level in *miR-373* mimics or sh*SIRT1*-transfected PC cells. PC; Pancreatic cancer, *; P<0.05, **; P<0.01, ROS; Reactive oxygen species, MDA; Malondialdehyde, and SOD; Superoxide dismutase.

Since SIRT1 was associated with alteration in the PGC-1α/NRF2 axis, the effects of *miR-373* overexpression or *SIRT1* silencing on PGC-1α/NRF2 signaling pathway were studied. Western blot analysis indicated that the level of PGC-1α, NRF2 and eNOS were dramatically decreased in *miR-373* mimics and sh*SIRT1*-transfected PC cells. In contrast, protein accumulation of iNOS was increased significantly in these *miR-373* mimics and sh*SIRT1*-transfected PC cells (Fig .5A-D). 

Since oxidative stress response was an early event in cell apoptosis associated with PGC-1α/ NRF2 signaling pathway, the ROS, MDA and SOD levels involved in *miR-373* and *SIRT1*-mediated regulation were assessed in the PC cells. The contents of ROS and MDA were significantly increased in PC cell lines after transfection with *miR-373* mimics or sh*SIRT1* in comparison with the control groups (Fig .5E, F), whereas, the level of SOD was dramatically decreased by transfection of *miR-373* mimics or sh*SIRT1* in the AsPC-1 and PANC-1 cells (Fig .5G). These results suggested that accumulation of *miR-373* or silence of *SIRT1* could lead to the activation of oxidative stress response via suppressing PGC-1α/NRF2 pathway in both of the PC cells.

### *miR-373* mediates proliferation and apoptosis of pancreatic cancer cells by SIRT1/PGC-1α/NRF2 signaling pathway

**Fig.6:**
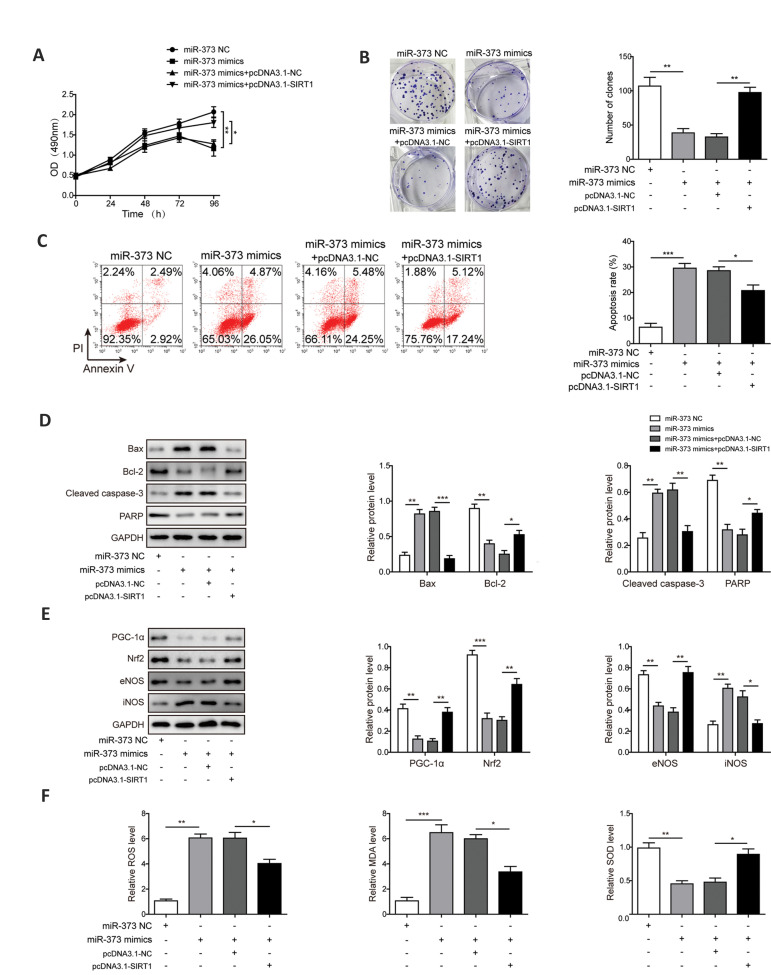
*miR-373* mediates proliferation and apoptosis of PC cells by *SIRT1*/PGC-1α/NRF2 axis. **A. **Cell proliferation was evaluated by the MTT assays. **B.** Number of cell colonies was tested by colony formation assay. **C. **Cell apoptosis was tested via Annexin-V/PI staining of flow cytometry analysis. **D. **Western blotting of apoptosis-related proteins BCL-2, BAX, cleaved CASPASE-3 and PARP. **E.** Western blotting of PGC-1α, NRF2, eNOS and iNOS proteins.** F.** Relative levels of ROS, MDA and SOD. AsPC-1 cells were treated with *miR-373* NC, *miR-373* mimics, *miR-373* mimics+pcDNA3.1-NC, and *miR-373* mimics+pcDNA3.1-*SIRT1*. PC; Pancreatic cancer, *; P<0.05, **; P<0.01, ***; P<0.001, ROS; Reactive oxygen species, MDA; Malondialdehyde, and SOD; Superoxide dismutase.

To elucidate whether the SIRT1/PGC-1α/NRF2 signaling pathway participates in the regulatory mechanism of *miR-373* in PC, AsPC-1 cells were treated with *miR-373* mimics followed by pcDNA3.1-*SIRT1*. As shown in Figure 6A and B, MTT and colony formation assays showed no pronounced difference regarding proliferation of PC cells between *miR-373* mimics and *miR-373* mimics+pcDNA3.1-NC groups. We found reduction of proliferative ability of PC cells by *miR-373* mimics was enhanced by overexpression of SIRT1. The results of apoptosis analysis also showed no significant difference of cell apoptosis between *miR-373* mimics and *miR-373* mimics+pcDNA3.1-NC groups, and overexpression of SIRT1 inhibited cell apoptosis induced by *miR-373* (Fig .6C). Western blot analysis displayed increased protein expressions of BAX and cleaved CASPASE-3, while the protein levels of BCL-2 and uncleaved PARP were reduced in PC cells treated with *miR-373* mimics. In contrast, overexpression of SIRT1 reversed *miR-373* mimics-induced effects (Fig .6D). Subsequently, western blot analysis also showed that transfection of pcDNA3.1-*SIRT1* increased protein levels of PGC-1α, NRF2, eNOS and decreased protein level of iNOS in *miR-373* mimics-treated PC cells, suggesting that overexpression of SIRT1 reversed effects of *miR-373* on PGC-1α/NRF2 signaling and oxidative stress response (Fig .6E). As depicted in Figure 6F, no significant difference concerning the relative levels of ROS, MDA and SOD in *miR-373* mimics as well as *miR-373* mimics+pcDNA3.1- NC groups was observed. We found that reduced levels of ROS and MDA and increased level of SOD in the presence of *miR-373* mimics were rescued by overexpression of SIRT1. Overall, the results suggested that *miR-373* inhibited PC cell proliferation but accelerated apoptosis through modulating oxidative stress response via SIRT1/PGC-1α/NRF2 axis.

## Discussion

PC is a lethal malignancy characterized by aggressive biological behaviors with the pronounced potential for invasion and metastasis, as well as resistance to many available anti-cancer agents (25, 26). PC is generally diagnosed in the more advanced stages, with a scarcity of effective therapies available. The present study demonstrated that *SIRT1* was verified as the direct target of *miR-373* in PC cells. Overexpression of *miR-373* decreased cellular accumulation of SIRT1 and resulted in the suppression of SIRT1-mediated PGC-1α/NRF2 signaling pathways. The downstream oxidative response was enhanced by this mechanism, which hindered the progression of PC by impairing PC cells proliferation in one hand and enhancing apoptosis of PC cells on the other hand. The roles of *miR-373*/*SIRT1* axis on regulating proliferation and apoptosis in PC cells were studied for the first time. Our findings provided the possibility that *miR-373* might serve as a potential new therapy for PC. 

In our work, we proved that overexpression of *miR-373* in AsPC-1 and PANC-1 cells not only caused suppression of cell proliferation, but also boosted cell apoptosis. These two effects, together, would result in inhibition of PC development. In combination with the reality that *miR-373* level was dramatically declined in PC, we concluded that *miR-373* was indeed a tumor suppressor. Silence of *SIRT1* also had similar effects on proliferation and apoptosis in the examined PC cells. In our study, we also found that application of miR-373mimics or sh*SIRT1* in PC cells could stimulate activation of apoptosis through up-regulating expression of BAX and cleaved CASPASE-8/9/3, while down-regulating expression of BCL-2 and uncleaved PARP. Researches proved that *miR-373* had significant regulating functions in breast cancer and seminoma (27, 28). In recent research, the role of a *miR-373* family member (*miR-373-3p*) in cell growth of lung adenocarcinoma was profiled, by targeting amyloid precursor protein (29). However, the function and mechanism of *miR-373* regulating PC progression have not been much clarified. Zhang et al. (30) reported that *miR-373* could down-regulate expression levels of TP53INP1, LATS2 and CD44 to promote PC development. Shao et al. (31) proved that LATS2 reduced antioxidant protein levels which in turn promoted the oxidative stress. These studies suggested that *miR-373* played important roles in promoting development of pancreatic cancer. However, we found that *miR-373* could inhibit cell proliferation and apoptosis via regulation of SIRT1/PGC-1α/NRF2 axis in pancreatic cancer, which was consistent with the report of Nakata et al. (13) indicating that *miR-373* was down-regulated in PC and suppressed invasion of tumor cells. Furthermore, Hua et al. reported that low level of serum *miR-373* predicted poor prognosis in patients with PC (14). This was also consistent with our study. This controversial conclusion needs to be further studied in future. Additionally, interaction of *miR-373* with LATS2 in PC is deeply worthy to explore and investigate, in our lab in future.

Subsequently, we elucidated whether SIRT1/PGC-1α/ NRF2 pathway participated in the regulatory mechanism of *miR-373* in PC cells. PC cells were treated with *miR-373* mimics followed by pcDNA3.1-*SIRT1*. Our results suggested overexpression of SIRT1 reversed pro-apoptotic an anti-proliferative effects of *miR-373* on PC cells. At the same time, inhibition of PGC-1α/NRF2 signaling pathway mediated by *miR-373* mimics was weakened in the presence of pcDNA3.1-*SIRT1*. Dual-luciferase reporter assay proved that *miR-373* exerted its regulating functions by direct interaction with *SIRT1*. The present work was a novel report about *miR-373* negatively regulating *SIRT1* interfered in PC progression. Liu and Wilson (32) discovered that *miR-373* was able to target the 3´-UTR of *mTOR* and *SIRT1* mRNA to regulate MMP-9 expression in fibrosarcoma HT1080 cells. In addition, *miR-373* was also found to up-regulate MMP-9 by regulating *SIRT1*, leading to activation of the RAS/ RAF/MEK/ERK pathways in fibrosarcoma cells (33). Similarly, *SIRT1* was identified and proved as a direct target of *miR-373* in PC cells for the first time, in the present work. Taken together, this conserved targeting pattern and blocking *SIRT1* expression by *miR-373* suggested that regulatory mechanisms of MMP-9, mediated by *miR-373*, may also be existed in PC cells. This could be studied in our future work. In the previous work, *miR-373* was also indicated as a potential “onco-miRNA” in other multiple cancers (34). 

SIRT1 plays important roles in diverse cellular processes including oxidative stress alleviation, oncogenesis, aging and cancer progression (35). PGC-1α, as one of downstream targets of SIRT1, is not only a regulator of mitochondrial genesis, but also known to protect from oxidative stress. Previous studies observed that SIRT1/PGC-1α/NRF2 signaling pathway was correlated with various cellular responses to oxidative stress. For instance, overexpression of SIRT1 strongly induced PGC-1α/NRF2 expression levels in human cancer cells (36). In our study, the enhanced oxidative metabolism was found in the *miR-373* mimics or sh*SIRT1*-transfected PC cells. Here, we also found that *miR-373* mimics or sh*SIRT1* could disturb expressions of PGC-1α and NRF2, while up-regulate ROS and MDA levels. These results indicated that overexpression of *miR-373* could interrupt SIRT1- mediated activation of PGC-1α/NRF2 pathway, thus enhancing oxidative stress, preventing PC progression. A large number of studies have also shown that ROS can stimulate tumorigenesis via oxidation of DNA and subsequent mutation of genes promoting carcinogenesis (37). ROS production has been detected in various cancers, which has been proven to have various roles. For example, ROS can activate pro-tumourigenic signaling, promote cell survival and proliferation, in addition to driving DNA damage and genetic instability (38). DeNicola et al. (39) also reported activating a ROS-detoxification program contributed to tumorigenesis. In contrast, Li et al. (40) found that cardamonin suppressed tumor growth by inducing G2/M phase cell cycle arrest and apoptosis via upregulation of ROS. These studies suggested that ROS could also promote anti-tumourigenic signaling, which were consistent to our study. To summarize, this is a novel research that studied *miR-373*/*SIRT1* axis and PGC-1α/ NRF2 pathway-mediated oxidative stress in the PC cell proliferation and apoptosis. 

## Conclusion

To conclude, our observation demonstrated that *miR-373* was revealed to participate in the modulation of apoptosis and proliferation by directly targeting *SIRT1* in PC cells for the first time. Moreover, the novel correlation of *miR-373*/*SIRT1* axis with PGC-1α/NRF2 pathway to regulate proliferation and apoptosis of PC cells was studied. Our results suggested *miR-373* might be a potential drug target for PC treatment. 
